# Integrated Proteomic and Metabolomic Analysis of the Testes Characterizes BDE-47-Induced Reproductive Toxicity in Mice

**DOI:** 10.3390/biom11060821

**Published:** 2021-05-31

**Authors:** Liang Xu, Songyan Gao, Hongxia Zhao, Liupeng Wang, Yiyi Cao, Jing Xi, Xinyu Zhang, Xin Dong, Yang Luan

**Affiliations:** 1 School of Public Health, Hongqiao International Institute of Medicine, Shanghai Jiao Tong University School of Medicine, Shanghai 200025, China; jack@ibsbio.com (L.X.); wlpzeus@163.com (L.W.); yycao@shsmu.edu.cn (Y.C.); xijing@shsmu.edu.cn (J.X.); xyzhang999@shsmu.edu.cn (X.Z.); 2 Shanghai Integrated Biotech Solutions Co., Ltd., Shanghai 201100, China; 3 Institute of Translational Medicine, Shanghai University, Shanghai 200444, China; sy_gaosmmu@163.com; 4 School of Medicine, Shanghai University, Shanghai 200444, China; honsha65678792@163.com

**Keywords:** BDE-47, integrated proteomic and metabolomic analysis, male reproductive toxicity, *MYC*

## Abstract

A representative congener of polybrominated diphenyl ethers in the environment, 2,2′,4,4′-tetrabromodiphenyl ether (BDE-47), is associated with male reproductive toxicity, yet the underlying mechanisms remain largely unclear. In this study, mice were administered environmentally relevant concentrations of BDE-47 for six weeks. Histopathological observations showed that BDE-47 induced inflammatory reactions and damaged the testes. By conducting an integrated proteomic and metabolomic analysis coupled with a bioinformatic analysis using ingenuity pathway analysis (IPA) methods, we found that BDE-47 mainly affected the molecules involved in free radical scavenging, cell death and survival, neurological disease, and inflammatory response. IPA canonical pathways showed inflammatory and apoptosis pathways, including hepatic fibrosis/hepatic stellate cell activation, the GP6 signaling pathway, tight junction signaling, acute phase response signaling, LXR/RXR activation, unfolded protein response, and FXR/RXR activation, which are related to male reproductive toxicity. Key transcriptional regulator networks were activated via a focus on upstream regulator analysis. The expression of *MYC* and Clu as the core transcriptional factor and targeted protein, respectively, was verified. It is further proposed that *MYC* may contribute to the etiology of male reproductive toxicity. These findings will improve our understanding of the mechanisms responsible for BDE-47-induced male reproductive toxicity, which may promote the discovery of useful biomarkers indicative of BDE-47 exposure.

## 1. Introduction

Polybrominated diphenyl ethers (PBDEs) are flame retardants widely used in various commercial products. As PBDEs are lipophilic and persistent, PBDE congeners have accumulated in the environment and biota over recent decades [1,2,3,4]. As a result, PBDE accumulation in humans has increased exponentially [1,5]. PBDEs are of increasing concern owing to their endocrine disruption effects, neurodevelopmental toxicity, and potential carcinogenicity [6,7,8]. Based on the physicochemical properties and health effects of PBDEs, commercial penta-, octa-, and deca-PBDE mixtures have been declared persistent organic pollutants under the Stockholm Convention. In particular, 2,2′,4,4′-tetrabromodiphenyl ether (BDE-47) is a major component of commercial penta-PBDE. Environmentally and biologically, BDE-47 exhibits relatively higher detection rates and concentrations, as well as a higher toxicity than elevated PBDE congeners [9,10,11]. Considering its exposure and toxicity potential, BDE-47 may significantly contribute to the toxicity associated with PBDE exposure. BDE-47 is currently being phased out from markets, but it can be released from consumer products manufactured prior to restrictions and generated by the debromination of higher PBDE congeners [5,6]. Thus, BDE-47 exposure is likely to persist, such that the associated health risks should be given priority.

BDE-47 has reproductive effects in both humans and animals. Population studies have shown that BDE-47 exposure is negatively associated with sperm mobility and may even have long-term effects on pregnancy in females [12,13]. In male rodents, BDE-47 causes impaired sperm motility and capacitation, increased abnormal spermatozoa, reduced spermatogenesis, and smaller testes in mice or rats [12,14,15,16]. BDE-47-induced germ cell apoptosis was observed in both male mice and rats, which is likely a mechanism of its reproductive toxicity [14,15,16,17]. PBDEs can also disrupt the homeostasis of thyroid and sex hormones; furthermore, evidence has linked the endocrine disruption of PBDEs to its reproductive toxicity [12,18,19]. However, previous studies have not yet fully elucidated the underlying molecular mechanisms of these adverse reproductive outcomes.

Proteomics and metabolomics, as important branches of systems biology, have high integrity and are not limited to a single protein or metabolite. These disciplines incorporate all components of a biological system to build a network of mutual relations among the components [20,21]. In addition, proteomics and metabolomics can reveal the response of an organism to external stimuli on a global profile, such as poisons and diseases. Compared with genetic research, proteomics can better explain changes in the mechanisms of physiological and pathological states; however, it is more challenging to analyse the proteome due to posttranscriptional and posttranslational modifications, and protein–protein interactions [22]. Therefore, proteomics aids the study of toxic reaction mechanisms to environmental pollutant exposure. Owing to their diversity and complexity, proteins have proven difficult to investigate by conventional techniques [23]. Quantitative proteomics, such as an isobaric tag for relative and absolute quantitation (iTRAQ) technology based on liquid chromatography-mass spectrometry (LC-MS), has greatly improved the throughput, sensitivity, and accuracy of proteomic research [24].

Metabolomics mainly focuses on small molecular metabolites downstream of life activities and more directly reflects the physiological or pathological states of an organism [21,25]. The biological information for organisms flows from proteins to metabolites. The integration of metabolomics and proteomics provides a systematic view of systems biology, clarifying the metabolic alterations caused by certain proteins. Consequently, we can elucidate the toxicity reaction mechanism of the exposure of an organism to environmental pollutants.

To our knowledge, no existing study has integrated proteomics and metabolomics to study male reproductive toxicity induced by BDE-47. In this study, iTRAQ-based proteomic and ultra-performance LC-MS-based metabolomic approaches were employed to investigate changes in the proteomic and metabolomic profiles of mouse testes following exposure to BDE-47. Moreover, the signaling pathways involved in BDE-47 action were analyzed. The results of this study provide insights into the mechanisms underlying BDE-47-induced male reproductive toxicity and can aid in the development of potential biomarkers for health risk assessments of environmental BDE-47 exposure.

## 2. Materials and Methods

### 2.1. Animals and Treatments

C57BL/6J gpt delta mice were obtained from the National Institutes of Health Sciences, Japan. The animals were bred and housed in pathogen-free rooms with a 12/12 h light/dark cycle at a temperature of 20–26 °C and humidity of 30–70%. All animal experiments were approved by the National Institutes of Health Guide for the Care and Use of Laboratory Animals with the ethical clearance (SYXK-2013-0050) of the Scientific Investigation Board of Shanghai Jiao Tong University School of Medicine, Shanghai, China. All experiments followed the guidelines and regulations of the Shanghai Jiao Tong University School of Medicine.

**Figure 1:**
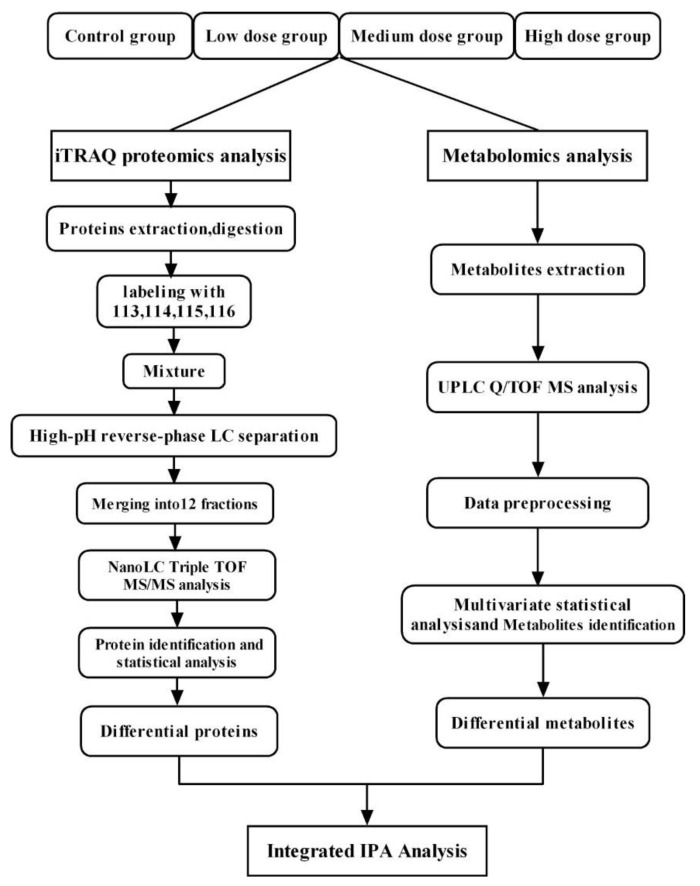
Flowchart of integrated analysis strategy for BDE-47-induced reproductive toxicity.

Male mice (7 to 8 weeks old) were randomized by body weight and allocated to four treatment groups (n = 6 mice per group). After 1 week of acclimatization, BDE-47 (CAS: 5436-43-1, Matrix Scientific, SC, USA) was administered to the animals at doses of 0, 1.5, 10, and 30 mg/kg/d in soybean oil via gavage 6 days per week for 6 weeks. The mice could access food and water ad libitum. Twenty-four hours after the last treatment, the mice were anesthetized and euthanized by cervical dislocation. The bilateral testis and epididymis were subsequently dissected. The tissues were either fixed for histological analysis or frozen in liquid nitrogen and stored at −80 °C for metabolomic and proteomic analyses (Figure 1).

### 2.2. Histological Analysis

Cross section of unilater testes and epididymides from each animals were trimmed and collected, and fixed in modified Davidson solution and 10% neutral formalin fixative, respectively. After fixation, the tissues were dehydrated with graded ethanol, vitrification by dimethylbenzene, embedded in paraffin and cut into 3 µm sections. The sections were stained with hematoxylin-eosin. All samples were examined manually under a ZEISS AXIO microscope (Zeiss, Berlin, Germany), and images were taken using an Aperio Scan ScopeXT slice scanning system (Leica, Wetzlar, Germany) with a 20× objective.

### 2.3. iTRAQ-Based Quantitative Proteomic Analysis

The iTRAQ-based quantitative proteomic analysis was based on our laboratory protocol [26]. Three random tissue samples from each treatment group were pooled. Proteins from the four pooled samples were extracted, purified, and quantified using bicinchoninic acid. A total of 120 μg of protein from each pooled sample was reduced and alkylated using the Reducing and Cysteine-Blocking Reagents in the iTRAQ buffer kit (SCIEX Inc., Framingham, MA, USA.), after which it was digested overnight with sequencing-grade trypsin (1:50 (w:w)) at 37 °C. The resulting tryptic peptides were collected and labeled with iTRAQ^®^ reagents (AB SCIEX Inc., Framingham, MA, USA) according to the manufacturer’s instructions.

The tryptic peptides of the control group (0 mg/kg), low dose group (1.5 mg/kg), medium dose group (15 mg/kg), and high-dose group (30 mg/kg) were labeled with tags of 113, 114, 115, and 116, respectively. The labeled peptides were mixed, dried, and re-dissolved in 100 µL of 20 mM ammonium formate (pH = 10). The peptide mixture was fractioned via high-pH reverse-phase LC using a ZORBAX 300Extend-C18 (4.6 × 250 mm 5-micron; Agilent, Santa Clara, CA, USA) chromatographic column. All reverse-phase high-performance LC fractions were dried, re-dissolved, and merged into 12 fractions with 50 μL of 2% acetonitrile (ACN) and 0.1% formic acid (FA). These fractions were analyzed via LC-tandem MS (Eksigent Nano LC-Ultra System, Tandem TripleTOFTM5600+, AB SCIEX). The trap column was a ChromXP C18 (ChromXP C18_CL; 3 μm, 120 A, 350 μm × 0.5 mm) (Eksigent, Dublin, CA, USA). The flow rate was 3 μL/min and the loading time was 15 min. The analytical column was a C18 reversed-phase column (0.075 × 150 mm, 3 μm, 120 A). Mobile phase A consisted of 98% water, 2% ACN, and 0.1% FA. Mobile phase B comprised 98% ACN, 2% water, and 0.1% FA at a flow rate of 300 nL/min, with the following elution gradients: 0–0.1 min, 5–8% B; 0.1–60 min, 8–25% B; 60–75 min, 25–50% B; 75–75.5 min, 50–80% B; 75.5–80 min, 80% B; 80–80.5 min, 5–80% B; and 80.5–90 min, 5% B. The main MS parameters were electrospray ionization positive mode, an MS1 range of 350–1250 *m*/*z*, and a scanning time of 0.25 s. A maximum of 40 precursors per cycle was chosen for fragmentation from each MS spectrum, with a 0.1 s minimum accumulation time for each precursor and a dynamic exclusion of 18 s. The MS2 mass range was set to 100–1500 *m*/*z*.

Proteins were identified and quantified using the ProteinPilot^TM^ 4.5 software (AB SCIEX, Framingham, MA, USA) with the Paragon database search algorithm. The reviewed mouse protein database was downloaded from UniProt (http://www.uniprot.org/) (accessed on 7 December 2019). At a false discovery rate of <1%, only proteins with at least one unique peptide (unused > 2) were considered credible, and those with a 1.3-fold change (up or down) with statistical significance (*p* < 0.05) were filtered as different proteins between two groups.

### 2.4. Metabolomic Analysis

Frozen testicular tissues were thawed and weighed. Subsequently, 500 μL of an 80% methanol solution was added to each tissue sample, after which it was homogenized at 70 Hz for 2 min and centrifuged at 13,000 rpm and 4 °C for 15 min. Then, 150 μL of the supernatant was placed in the injection vial for analysis. Aliquots of each pretreated supernatant were mixed and used as a quality control sample.

Ultra-high performance LC-quadrupole time-of-flight MS was performed using an Agilent 1290 Infinity LC system coupled to an Agilent 6538 accurate-mass quadrupole time-of-flight mass spectrometer. Chromatographic separations were performed at 25 °C using a XBridge^®^ BEH HILIC 2.5 analytical column (2.1 mm × 100 mm, 2.5 μm; Waters Corporation, Milford, MA, USA). The flow rate was 0.4 mL/min and the injection volume was 3 μL. The mobile phase consisted of 0.1% FA (A) and ACN modified with 0.1% FA (B). The gradient elution conditions were 95% B for 0–2 min, 95–65% B for 2–10 min, 65–50% B for 10–13 min, and 50% B for 13–15 min, after which the mobile phase was re-equilibrated for 5 min. The mass spectrometer was operated in electrospray ionization positive and negative modes. The capillary voltage was 4 kV in positive mode and 3.5 kV in negative mode, with a drying gas flow of 11 L/min and gas temperature of 350 °C. The nebulizer pressure was set at 45 psi. The fragmentor voltage was set at 120 V and the skimmer voltage at 60 V. Data were collected in centroid mode and the mass range was set at 50–1000 *m*/*z*. The collision energy for MS/MS analysis was set at 10, 20, and 40 eV.

### 2.5. Bioinformatics Analysis

The quantified differential protein and metabolites were analyzed using IPA (Qiagen, Redwood City, CA, USA) [27]. To elucidate toxicological mechanisms in mouse testes treated with BDE-47, the identification numbers and fold changes in the BDE-47-regulated proteins and metabolites were subjected to IPA core analysis. IPA core analysis helps assess canonical pathways, diseases and functions, upstream regulators, and signaling networks associated with reproductive toxicity. A *p* value < 0.05 (Fisher’s exact test) and a z score ≠ 0 were used as the thresholds to determine the significant disturbance pathways. A z score > 2 represents significant activation, and a z score < −2 represents significant inhibition.

### 2.6. Real-Time PCR

The mouse testes were collected in RNAlater (Ambion Inc., Austin, TX, USA), and the total RNA was extracted with a TIANGEN RNA Extraction Kit (Tiangen Biotech, Beijing, China). The total RNA was reverse transcribed into cDNA. Real time PCR was subsequently performed using a PrimeScript^®^ RT Reagent Kit (Takara, Dalian, China) and SYBR Green Master Mix reagents on a ABI7500 real-time PCR system (Applied Biosystems, Foster City, CA, USA) following a prespecified program (one cycle of denaturing 95 °C for 3 min, followed by 40 amplification cycles at 95 °C for 15 s and 60 °C for 30 s). β-actin was used as the reference gene for normalizing gene expression. Three replicates were performed for each sample. The fold changes were analyzed via the 2^ΔΔCT^ method. Table S3 describes all of the primer sets.

### 2.7. Statistical Analysis

The data analyses were performed with the SPSS software (version 18.0). One-way ANOVA and t-test were used to test significant differences between multiple groups. In this study, *p* < 0.05 was considered statistically significant.

## 3. Results

### 3.1. Histological Analysis

**Figure 2:**
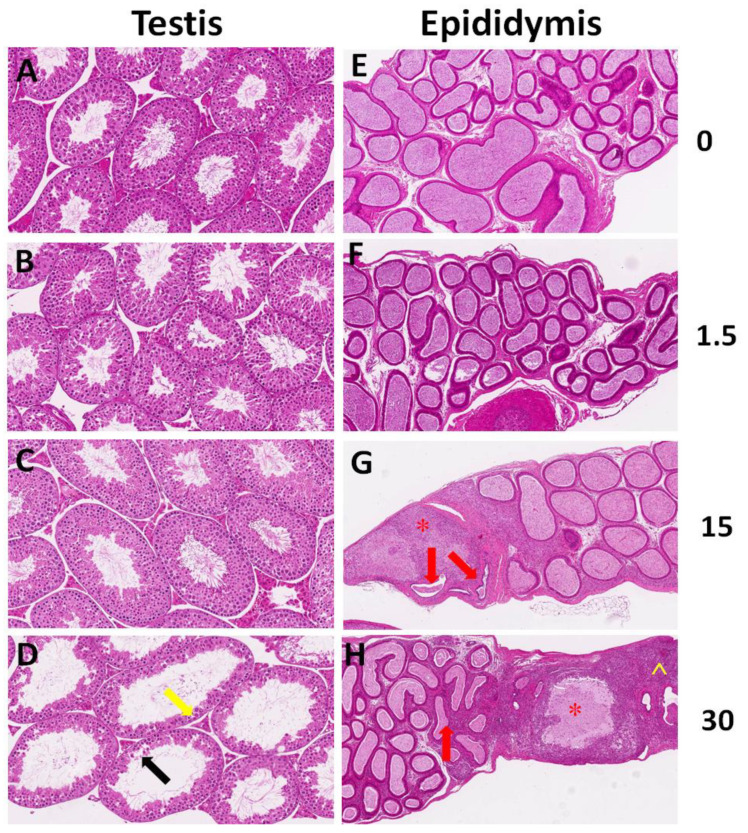
Histological analysis of the testes and epididymis of the BDE-47-treated mice. (**A**–**D**) Representative images of the testis sections. Black and orange arrows indicate necrotic spermatogenic cells and attenuated seminiferous epithelium, respectively. (**E**,**F**) Representative images of the epididymis sections. (**G**,**H**) Red arrows indicate reduced sperm in the epididymal lumens; asterisk (*) indicates spermatic granulomas; and carets (^) represent suppurative inflammation. Images were obtained at an original magnification of 20×.

Histological analysis was performed on both the testes and epididymis. Compared with those of the control group, the spermatogenic cells in the testes of the 30 mg/kg BDE-47-treated group suffered degeneration and necrosis accompanied by seminiferous epithelium thinning (Figure 2). No changes were observed in the testis in the lower dosage groups. Epididymis sections showed that BDE-47 caused sperm reductions in the epididymal lumens in the 10 and 30 mg/kg groups. In the former, spermatic granulomas were observed, whereas spermatic granulomas accompanied by suppurative inflammation were observed in the latter.

### 3.2. Proteome Analysis

**Figure 3:**
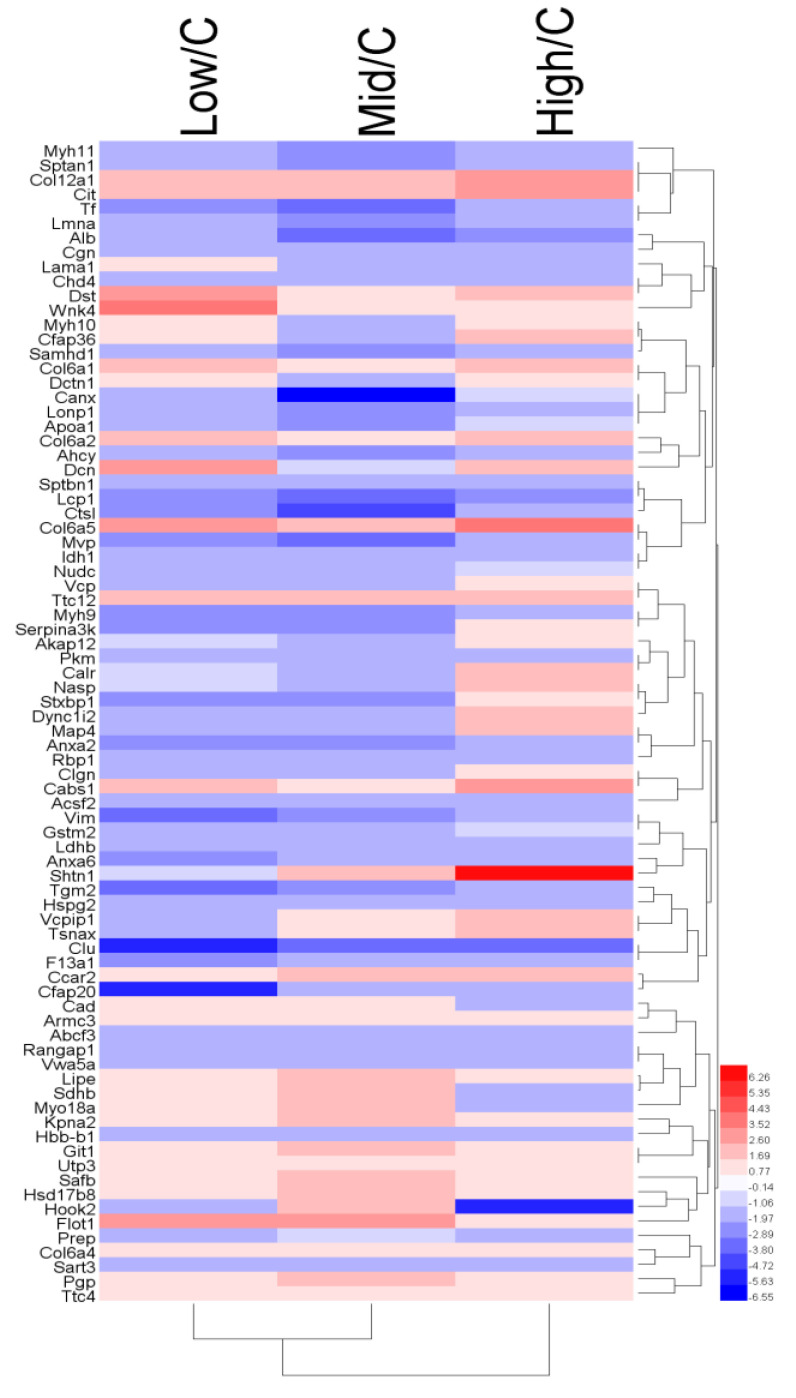
Hierarchical clustering showing the proteins across low, middle and high dose groups. *p* < 0.05.

We identified 29,709 peptides and 3834 highly reliable proteins (unused ≥ 2). According to the filtering criteria for the differential proteins in the control and BDE-47-treated groups (FC > 1.3 or < 0.77), 33 differential proteins were present in the low-dose and control groups, 12 of which were up-regulated and 21 down-regulated. In the medium-dose and control groups, 47 differential proteins were present, 15 of which were up-regulated and 32 down-regulated. The high-dose and control groups contained 31 differential proteins, 18 of which were up-regulated and 13 down-regulated. Table S1 lists the specific differential proteins. Hierarchical clustering analysis corroborated these results (Figure 3).

**Figure 4:**
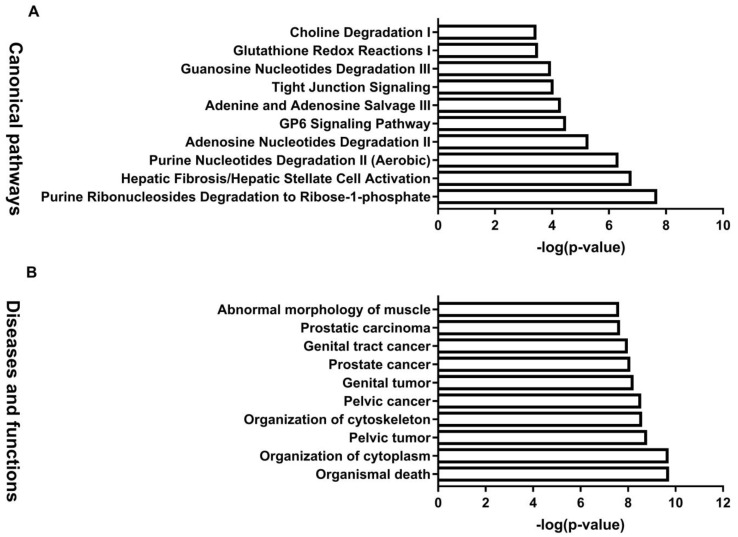
Canonical pathway, and diseases and functions analysis of the differentially expressed proteins. (**A**): Canonical pathway enrichment analysis for sets of the differentially expressed genes. (**B**): Diseases and functions enrichment analysis. A *p* value < 0.05 and an FDR value < 0.05 were selected as the significant criteria.

To examine the interactions among the proteins exhibiting an altered abundance in response to the BDE-47 treatments, we performed IPA analysis on the differentially expressed datasets. The differentially expressed proteins in response to BDE-47 in the testes were significantly enriched in the following pathways, which are related to male reproductive toxicity: Hepatic fibrosis/hepatic stellate cell activation, the GP6 signaling pathway, tight junction signaling, acute phase response signaling, LXR/RXR activation, unfolded protein response, and FXR/RXR activation (Figure 4A). Disease or function analysis of differentially expressed proteins indicates that differentially expressed proteins are mainly enriched in the abnormal morphology of the muscle, multiple tumors, cytoskeleton organization, organization of the cytoplasm, and organismal death (Figure 4B).

### 3.3. Metabolome Analysis

**Figure 5:**
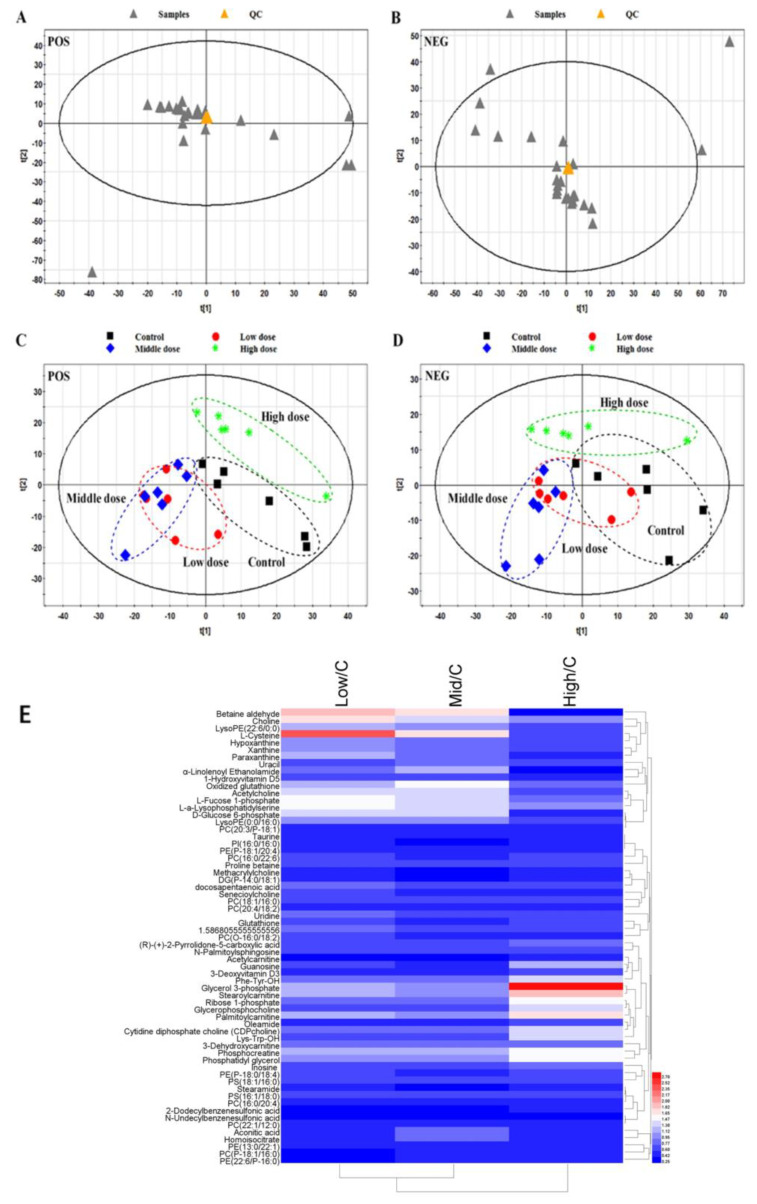
Score plots of the multivariate statistical analysis for the control group and 2,2′,4,4′-tetrabromodiphenyl ether (BDE-47)-treated groups (n = 6). Principal component analysis (PCA) score plot of the normal and quality control samples in (**A**) positive mode and (**B**) negative mode. Partial least squares discriminant analysis score plot for the control group and BDE-47-treated groups in (**C**) positive mode and (**D**) negative mode. (**E**) Hierarchical clustering of identified metabolites among the three groups (n = 6).

The raw data were collected according to the above LC-MS methods. The XCMS Package (https://metlin.scripps.edu/ (accessed on 7 December 2019) was used for peak extraction, alignment, and integration in the R software platform. We detected 1473 and 1175 features in the positive and negative modes, respectively. After weight normalization, the matrix was imported into SIMCA-P 11.0 for multivariate statistical analysis. Initially, unsupervised principal component analysis (PCA) was used to evaluate system stability. The quality control samples showed distinct clustering in both the positive and negative modes (Figure 5A,B), indicating that the LC-MS system was stable.

Furthermore, a supervised partial least squares discriminant analysis (PLS-DA) model was used to compare the control and three BDE-47-treated groups. According to the PLS-DA score plots (Figure 5C,D), the trends of the four groups differed. To identify the differential metabolites in testicular tissue after BDE-47 treatment, we performed a PLS-DA analysis for the control and treatment groups. Ions with Variable Importance in the Projection (VIP) values > 1 and *p* values < 0.05 were considered differential features. These ions were identified based on the laboratory methods [26]. Accurate molecular weights were matched with the METLIN database (https://metlin.scripps.edu/) (accessed on 7 December 2019) and Human Metabolome Database (http://www.hmdb.ca/) (accessed on 7 December 2019) The MS/MS spectra were compared with the Metlin MS/MS library or manually analyzed. Finally, 63 differential metabolites were identified (Figure 5E and Table S2). The heatmap of these metabolites was conducted using HemI 1.0 software [28].

### 3.4. Integrated IPA Analysis and MYC-Activation in BDE-47-Induced Male Reproductive Toxicity

**Figure 6:**
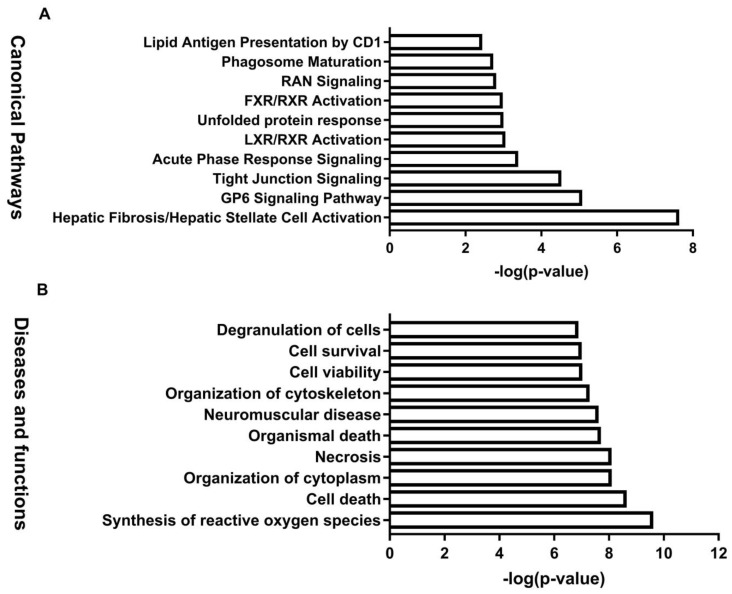
Canonical pathways, and diseases and functions analysis of the differentially expressed protein and metabolites. (**A**): Canonical pathway enrichment analysis for sets of the differentially expressed genes. (**B**): Diseases and functions enrichment analysis. *p* values < 0.05 and an FDR value < 0.05 were selected as the significant criteria.

IPA software was used to generate enrichment diagrams, elucidating the pathways and disease or function in mouse testes impacted by BDE-47. For an integrated proteomic and metabolomic analysis of the testes, 80 differential proteins and 63 modulated metabolites were subjected to IPA core analysis. The top 10 canonical pathways (Figure 6A) and top 10 diseases or functions (Figure 6B) were applied to evaluate the signaling pathway and toxicity predictions. The top 10 canonical pathways were mainly involved in regulation nucleotide metabolism and related pathways, including purine ribonucleosides degradation to ribose-1-phosphate, hepatic fibrosis/hepatic stellate cell activation, purine nucleotide degradation II (aerobic), adenosine nucleotide degradation II, the GP6 signaling pathway, adenine and adenosine salvage III, tight junction signaling, guanosine nucleotide degradation III, glutathione redox reactions I, and choline degradation I. The top 10 diseases or functions were related to free radical scavenging, cell death, and survival, including synthesis of reactive oxygen species, cell death, organization of the cytoplasm, necrosis, organismal death, neuromuscular disease, organization of the cytoskeleton, cell viability, cell survival, and degranulation of cells. Networks of differential proteins and metabolites that interacted biologically were generated to elucidate key regulators and their critical roles in BDE-47-induced male reproductive toxicity. The top seven networks with a score of ≥15 were identified (Table S4). Interestingly, free radical scavenging of top diseases and functions is one of the top two networks. IPA core analysis has an upstream regulator analysis function that allows the identification of the cascade in the upstream transcriptional regulators, which can predict which transcriptional regulators are involved in BDE-47-induced male reproductive toxicity and whether they are likely activated or inhibited. The analysis of top upstream regulator molecules showed that *MYC* is one of the top five. It has a z score > 2 and was activated (Figure 6A). Target molecules of *MYC* in the dataset have 10 molecules, namely ABCF3, AHCY, AKAP12, ALB, ANXA6, CAD, CANX, CLU, CTSV, and IDH1.

**Figure 7:**
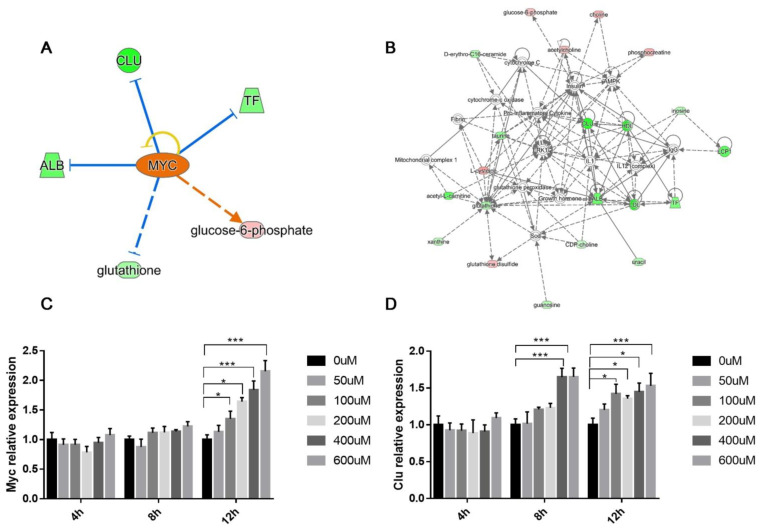
MYC-activation involved in 2,2′,4,4′-tetrabromodiphenyl ether (BDE-47)-induced male reproductive toxicity. (**A**) MYC was activated via Ingenuity Pathways Analysis (IPA). (**B**) Top-ranked gene network reconstructed from the genes associated with BDE-47-induced toxicity. (**C**) Clu and (**D**) *MYC* expression levels in the dose response and time course. β-actin was used as an equal loading control. Data are presented as mean ± SD of three independent experiments (n = 3). * *p* < 0.05, *** *p* < 0.01 significant differences compared to controls.

It seems that Alu and Clu were the key regulators in this network (Figure 7B), which may indicate their critical roles in BDE-47-induced male reproductive toxicity. The expression of *MYC* and Clu was verified via quantitative real-time PCR (Figure 7C,D).

## 4. Discussion

The major changes in the testes of the BDE-47-treated groups were degeneration and necrosis of the seminiferous epithelium, sloughing of dead and degenerating spermatogenic and Sertoli cells, and necrotic tubule collapse. BDE-47 had noxious effects on spermatogenic cells, including spermatogonia, primary spermatocytes, and round spermatids. Interestingly, BDE-47-induced changes in the epididymal sperm content occurred without changes in sperm production and morphology. In general, changes in the epididymal epithelium were not dramatic, but sperm disintegrated in the lumen, principal cells, and clear cells, which accumulated in structures identified as lysosomes. The leukocyte numbers and forms in the epididymal epithelium and interstitium changed, and areas of apparent inflammation appeared in the connective tissue surrounding the epididymal duct, sometimes accompanied by spermatic granulomas.

In this study, proteomic and metabolomic analyses showed the biological changes in the protein and metabolite profiles of the BDE-47-treated testicular tissue. The differentially expressed proteins in the testes in response to BDE-47 exposure were significantly enriched in the following pathways: Hepatic fibrosis/hepatic stellate cell activation, GP6 signaling pathway, tight junction signaling, acute phase response signaling, LXR/RXR activation, unfolded protein response, and FXR/RXR activation. With the development of global profiling technologies, omics-based methods are increasingly used to identify the critical proteins, metabolites, and pathways involved in biological systems and their responses to environmental stresses. The application of toxicoproteomics and toxicometabolomics can improve our assessment of the toxicity mechanisms associated with BDE-47 exposure [29]. Therefore, changes of the protein and metabolic profiles in mouse testes exposed to BDE-47 were investigated using an integrated proteomic and metabolomic approach in this study. Differential proteins and metabolites, perhaps closely related to male reproduction, were identified. Disturbances from free radical scavenging, cell death and survival, cellular assembly and organization, cellular function, and the maintenance of collagen fiber organization around epithelial structures may be major factors contributing to BDE-47-mediated male reproductive toxicity. Decreased collagen fiber, thinning, and basal laminar disorders reduce the intracellular spaces between Sertoli cells and spermatogonia [30]. *Lama1*, which encodes a laminin alpha chain, is involved in the basement membrane of the seminiferous epithelium and is required for normal testicular function [31]; down-regulation of the *Lama1* endogenous expression may damage testicular function. *Col6a2* plays a major role in cell adhesion and affects the secretion of testicular Leydig cells. COL6A2 is associated with infertility in human Azoospermia and involved in male reproductive disorders [32]. Up-regulation of *Col6a2* may interrupt epididymal homeostasis.

We identified c-myc as the key transcriptional factor through IPA upstream regulator analysis. c-Myc is a proto-oncogene and a strong pleiotropic transcription factor known to coordinate cell cycle growth, progression, and apoptosis. Its expression was detected in situ during spermatogenesis in adult mouse testes [33]. Some studies on environmental chemicals with reproductive toxicity have revealed that c-Myc protein response to gossypol exposure and spermatocyte apoptosis induced by gossypol is correlated with c-Myc protein expression [34]. c-Myc is involved in spermatogenesis, which might be mediated by cell cycle regulation and the increased cell apoptosis of sertoli cells [35]. Recent studies revealed that some microRNAs are involved in c-Myc-associated male reproductive toxicity, the roles of bta-miR-34b, and miR-362 in cell proliferation, while the apoptosis of immature Sertoli cells have also been reported [36,37]). Moreover, c-Myc participates in FSH induction of various genes of relevance in cell cycle progression [38]. Myc-mediated glycolysis is an important factor that increases the frequency of spermatogonial stem cells’ self-renewal division [39].

We demonstrated that exposure to BDE-47 caused Clu mRNA changes in mouse testes, accompanied by changes in Alb levels, with the maximum change observed at 72 h after treatment. Therefore, Clu appears to form a part of the cellular response to oxidative stress. Clu may act as an extracellular molecular chaperone, scavenging extracellular misfolded or denatured proteins caused by stress-induced injury [40,41]. While further studies are required to explore the causal association between *MYC* and the identified proteins and metabolites, in this study, an integrated proteomic and metabolomic approach was used to obtain new insights into the molecular mechanisms underlying BDE-47-induced male reproductive toxicity.

## 5. Conclusions

BDE-47 is a representative congener of PBDEs in the environment and is known to have reproductive toxicity, but the underlying mechanisms remain to be clarified. In the present study, we conducted an integrated proteomic and metabolomic analysis coupled with a bioinformatic analysis of IPA methods to improve the understanding of the mechanisms responsible for BDE-47-induced male reproductive toxicity. Histological analysis on the testes and epididymis indicated that BED-47 causes significant testicular damage and epididymal suppurative inflammation. Histological analysis on the testes and epididymis indicated that BED-47 causes significant testicular damage and epididymal suppurative inflammation. We used iTRAQ-based proteomic and ultra-performance liquid chromatography–mass spectrometry-based metabolomic approaches to investigate changes in the proteomic and metabolomic profiles of mouse testes following BDE-47 exposure at doses of 0, 1.5, 10, and 30 mg/kg/d. We identified the critical proteins, metabolites, and pathways involved in a reproductive system affected by and responding to BDE-47 using global profiling technologies, toxicoproteomics, and toxicometabolomics. To our knowledge, this is the first study to integrate proteomics and metabolomics to study male reproductive toxicity induced by BDE-47 and it provided a novel view into BDE-47-induced male reproductive toxicity. Based on upstream analysis and validation at the mRNA level, we speculate that *MYC* may promote BDE-47-induced reproductive toxicity by targeting CLU proteins. However, more studies that are molecular based are needed to determine its specific downstream pathway. To our knowledge, this is the first study to integrate proteomics and metabolomics to study male reproductive toxicity induced by BDE-47, and we provided a novel view into its toxic mechanism.

## Data Availability

All data are given in the manuscript and the supplementary data.

## Supplementary Materials

The following are available online at https://www.mdpi.com/article/10.3390/biom11060821/s1, Table S1: Significantly differential proteins in testicular tissue between Control group and each dose of BDE47 group, Table S2: Significantly differential metabolites in testicular tissue between Control group and each dose of BDE47 group, Table S3: Quantitative real-time PCR (qRT-PCR) primer sequences for selected genes, Table S4: Top networks identified using Ingenuity Pathways Analysis software for significantly differential metabolites and proteins in testicular tissue.

## Abbreviations

ACN: acetonitrile; BDE-47: 2,2′,4,4′-tetrabromodiphenyl ether; FA: formic acid; IPA: Ingenuity Pathways Analysis; iTRAQ: isobaric tag for relative and absolute quantitation; PBDE: polybrominated diphenyl ether; PLS-DA: partial least squares discriminant analysis.
