# Supplementary material for: Integrated Proteomic and Metabolomic Analysis of the Testes Characterizes BDE-47-Induced Reproductive Toxicity in Mice
Source: Biomolecules. 2021 May 31;11(6):821. doi: 10.3390/biom11060821 (PMC8229108; doi:10.3390/biom11060821)
Supplement: Supplementary file 1 [file biomolecules-11-00821-s001.zip › biomolecules-1231306-supplementary.pdf]

Table S1. Significantly differential proteins in testicular tissue between Control group and each dose of BDE47 group.

No.	Uniprot Accession No.	Gene name	Protein name	Low/C		Mid/C		High/C	
				FC	p	FC	p	FC	p
1	O08638	Myh11	Myosin-11	-1.96	0.0018	-2.83	0.0000		
2	P16546	Sptan1	Spectrin alpha chain, non-erythrocytic 1	-1.69	0.0112	-2.05	0.0001		
3	P07724	Alb	Serum albumin	-1.84	0.0001	-3.63	0.0000	-2.73	0.0000
4	Q01853	Vcp	Transitional endoplasmic reticulum ATPase					1.50	0.0476
5	Q61879	Myh10	Myosin-10					1.46	0.0311
6	Q9WTQ5	Akap12	A-kinase anchor protein 12					1.57	0.0024
7	Q62261	Sptbn1	Spectrin beta chain, non-erythrocytic 1			-1.47	0.0268		
8	Q8VDD5	Myh9	Myosin-9			-2.42	0.0104		
9	P52480	Pkm	Pyruvate kinase PKM	-1.71	0.0461	-1.77	0.0035		
10	Q921I1	Tf	Serotransferrin	-2.31	0.0028	-2.94	0.0002	-1.61	0.0025
11	P20152	Vim	Vimentin	-3.50	0.0000	-2.42	0.0000		
12	Q99MD9	Nasp	Nuclear autoantigenic sperm protein					1.72	0.0086
13	B2RQC6	Cad	CAD protein	1.64	0.0161				
14	A6H584	Col6a5	Collagen alpha-5(VI) chain	3.25	0.0000	2.29	0.0137	3.70	0.0000
15	P52194	Clgn	Calmegin			-1.91	0.0377		
16	Q60847	Col12a1	Collagen alpha-1(XII) chain	2.44	0.0002			3.25	0.0000
17	Q9QUR6	Prep	Prolyl endopeptidase	-1.53	0.0275				
18	P27546	Map4	Microtubule-associated protein 4					1.75	0.0279
19	A2AX52	Col6a4	Collagen alpha-4(VI) chain			1.45	0.0297		
20	P14211	Calr	Calreticulin					2.11	0.0298
21	P21981	Tgm2	Protein-glutamine gamma-glutamyltransferase 2	-3.37	0.0037	-2.17	0.0177	-1.64	0.0351
22	Q04857	Col6a1	Collagen alpha-1(VI) chain	1.87	0.0006			1.89	0.0044
23	Q8VDP4	Ccar2	Cell cycle and apoptosis regulator protein 2			2.00	0.0171		
24	P14824	Anxa6	Annexin A6	-2.01	0.0044	-1.85	0.0080		
25	O08788	Dctn1	Dynactin subunit 1			-1.38	0.0437		
26	O88844	Idh1	Isocitrate dehydrogenase [NADP] cytoplasmic			-1.98	0.0457		

27	P07356	Anxa2	Annexin A2			-2.56	0.0177		
28	P35564	Canx	Calnexin			-6.55	0.0089		
29	Q02788	Col6a2	Collagen alpha-2(VI) chain	1.98	0.0026			1.82	0.0253
30	P50247	Ahcy	Adenosylhomocysteinase			-2.09	0.0414		
31	Q8CHP8	Pgp	Glycerol-3-phosphate phosphatase			2.36	0.0417		
32	P52293	Kpna2	Importin subunit alpha-1			1.87	0.0405		
33	Q9JLI8	Sart3	Squamous cell carcinoma antigen recognized by T-cells 3	-1.38	0.0141				
34	P19137	Lama1	Laminin subunit alpha-1			-1.63	0.0464	-1.49	0.0334
35	Q8C633	Cabs1	Calcium-binding and spermatid-specific protein 1	1.84	0.0246			3.28	0.0188
36	P59242	Cgn	Cingulin			-1.94	0.0174		
37	Q8C6E0	Cfap36	Cilia- and flagella-associated protein 36					1.77	0.0235
38	P46061	Rangap1	Ran GTPase-activating protein 1					-1.56	0.0345
39	P07759	Serpina3k	Serine protease inhibitor A3K	-2.40	0.0075	-2.21	0.0033		
40	Q8CDG3	Vcpip1	Deubiquitinating protein VCIP135					1.74	0.0075
41	P02088	Hbb-b1	Hemoglobin subunit beta-1			-1.34	0.0426	-1.87	0.0175
42	P16125	Ldhb	L-lactate dehydrogenase B chain	-1.82	0.0419	-1.54	0.0099		
43	Q05793	Hspg2	Basement membrane-specific heparan sulfate proteoglycan core protein	-1.92	0.0457				
44	Q06890	Clu	Clusterin	-4.79	0.0022	-3.66	0.0016	-2.96	0.0061
45	O88487	Dync1i2	Cytoplasmic dynein 1 intermediate chain 2					2.15	0.0164
46	Q6PDQ2	Chd4	Chromodomain-helicase-DNA-binding protein 4			-1.41	0.0455		
47	D3YXK2	Safb	Scaffold attachment factor B1			2.54	0.0280		
48	P48678	Lmna	Prelamin-A/C			-2.36	0.0475		
49	Q61233	Lcp1	Plastin-2	-2.83	0.0126	-3.66	0.0019	-2.19	0.0202
50	A2AU72	Armc3	Armadillo repeat-containing protein 3	1.54	0.0167	1.51	0.0057		
51	Q60710	Samhd1	Deoxynucleoside triphosphate triphosphohydrolase SAMHD1			-2.78	0.0434		
52	Q8VCW8	Acsf2	Acyl-CoA synthetase family member 2, mitochondrial	-1.64	0.0322				
53	Q68FF6	Git1	ARF GTPase-activating protein GIT1			1.79	0.0240		

54	Q8BW49	Ttc12	Tetratricopeptide repeat protein 12	1.74	0.0377			1.91	0.0265
55	Q8CGK3	Lonp1	Lon protease homolog, mitochondrial	-1.75	0.0037	-2.07	0.0052	-1.72	0.0082
56	P54310	Lipe	Hormone-sensitive lipase			1.72	0.0382		
57	Q9QZE7	Tsnax	Translin-associated protein X					1.89	0.0305
58	P15626	Gstm2	Glutathione S-transferase Mu 2	-1.72	0.0347				
59	O35685	Nudc	Nuclear migration protein nudC			-1.42	0.0294		
60	P06797	Ctsl	Cathepsin L1			-4.21	0.0328	-1.92	0.0373
61	Q00915	Rbp1	Retinol-binding protein 1			-1.92	0.0289		
62	Q91ZU6	Dst	Dystonin	2.63	0.0301				
63	Q9CQA3	Sdhb	Succinate dehydrogenase [ubiquinone] iron-sulfur subunit, mitochondrial					-1.80	0.0294
64	Q99KC8	Vwa5a	von Willebrand factor A domain-containing protein 5A					-1.64	0.0215
65	Q00623	Apoa1	Apolipoprotein A-I			-2.00	0.0229		
66	Q8K268	Abcf3	ATP-binding cassette sub-family F member 3					-1.82	0.0313
67	Q9EQK5	Mvp	Major vault protein			-3.60	0.0294		
68	P50171	Hsd17b8	Estradiol 17-beta-dehydrogenase 8			1.94	0.0249		
69	Q8BH61	F13a1	Coagulation factor XIII A chain	-2.47	0.0348				
70	Q8BTU1	Cfap20	Cilia- and flagella-associated protein 20	-5.40	0.0381				
71	O08599	Stxbp1	Syntaxin-binding protein 1	-2.29	0.0158				
72	P28654	Dcn	Decorin	2.61	0.0177				
73	Q80UE6	Wnk4	Serine/threonine-protein kinase WNK4	3.84	0.0447				
74	Q9JMH9	Myo18a	Unconventional myosin-XVIIIa			1.91	0.0460		
75	Q7TMK6	Hook2	Protein Hook homolog 2			1.94	0.0429		
76	P49025	Cit	Citron Rho-interacting kinase					2.86	0.0274
77	Q8K2Q9	Shtn1	Shootin-1					7.18	0.0337
78	Q8R3H9	Ttc4	Tetratricopeptide repeat protein 4			1.45	0.0298		
79	Q9JI13	Utp3	Something about silencing protein 10			1.37	0.0028		
80	O08917	Flot1	Flotillin-1	2.61	0.0445	3.28	0.0440		

Notes: Statistical differences ($p < 0.05$) between control and BED47-treated testes samples were determined by T-test.

Table 2. Significantly differential metabolites in testicular tissue between Control group and each dose of BDE47 group(n=6)

No.	Metabolite	CAS	RT(min)	Mass	Ion	Formula	Identification	Low/C		Mid/C		High/C	
								p	FC	p	FC	p	FC
1	Betaine aldehyde	7418-61-3	3.25	101.0841	[M+CH3OH+H]+	C5H11NO	MS	0.0034	1.94	0.0149	1.75	0.0235	0.31
2	Choline	62-49-7	4.30	103.0997	[M+H]+,[2M+H]+	C5H13NO	MS/MS	0.0014	1.65	0.0182	1.45	0.8115	1.04
3	Uracil	66-22-8	1.77	112.0273	[M+H]+	C4H4N2O2	MS	0.0630	0.76	0.1044	0.79	0.0042	0.60
4	L-Cysteine	52-90-4	7.11	121.0197	[M-H]-	C3H7NO2S	MS	0.0008	2.40	0.0592	1.71	0.4745	0.74
5	Taurine	107-35-7	6.87	125.0147	[2M+H]+	C2H7NO3S	MS	0.0186	0.56	0.0102	0.51	0.0204	0.56
6	(R)-(+)-2-Pyrrolidone-5-carboxylic acid	4042-36-8	1.74	129.0426	[M+H]+, [M-H]-,[2M-H]-,[M+FA-H]-	C5H7NO3	MS/MS	0.0232	0.73	0.0083	0.68	0.2020	0.86
7	Hypoxanthine	68-94-0	4.06	136.0385	[M-H]-	C5H4N4O	MS/MS	0.8479	0.98	0.1161	0.84	0.0016	0.65
8	Proline betaine	——	6.12	143.0946	[M+H]+, [M+K]+	C7H13NO2	MS	0.0017	0.67	0.0003	0.60	0.0013	0.66
9	3-Dehydroxycarnitine	——	4.47	145.1103	[M+H]+	C7H15NO2	MS	0.0046	0.78	0.0060	0.79	0.0233	0.83
10	Acetylcholine	51-84-3	2.14	145.1103	[M+H]+	C7H15NO2	MS	0.0142	1.45	0.0593	1.33	0.0728	0.69
11	Xanthine	69-89-6	4.62	152.0334	[M+H]+, [M-H]-	C5H4N4O2	MS/MS	0.7012	0.96	0.1378	0.86	0.0031	0.69
12	Glycerol 3-phosphate	57-03-4	8.13	172.0137	[M+H]+, [2M-H]-	C3H9O6P	MS	0.6437	1.27	0.9614	0.97	0.0042	2.87
13	Methacrylylcholine	——	1.26	172.1338	[M+Na]+	C9H18NO2	MS	0.0126	0.45	0.0041	0.34	0.0099	0.42
14	Aconitic acid	499-12-7	1.22	174.0164	[M+Cl]-	C6H6O6	MS	0.0272	0.55	0.3808	0.83	0.0134	0.49
15	Paraxanthine	611-59-6	7.04	180.0647	[M-H]-	C7H8N4O2	MS	0.4356	1.15	0.6960	0.92	0.0332	0.56

16	Senecioylcholine	——	1.11	186.1494	[M+Na]+	C10H20NO2	MS	0.0279	0.60	0.0044	0.46	0.0128	0.54
17	Acetylcarnitine	5080-50-2	3.27	203.1158	[M+H]+	C9H17NO4	MS/MS	0.0003	0.31	0.0001	0.25	0.0062	0.52
18	Homoisocitrate	3562-75-2	1.22	206.0427	[M+Cl]-	C7H10O7	MS	0.0228	0.56	0.3334	0.82	0.0161	0.53
19	Phosphocreatine	67-07-2	8.21	211.0358	[M+H]+	C4H10N3O5P	MS	0.2012	1.21	0.1338	1.24	0.0018	1.56
20	Ribose 1-phosphate	14075-00-4	8.80	230.0192	[M-H]-	C5H11O8P	MS/MS	0.4728	0.88	0.1932	0.78	0.0008	1.63
21	L-Fucose 1-phosphate	——	9.83	244.0348	[M+FA-H]-	C6H13O8P	MS	0.0085	1.49	0.0343	1.38	0.2980	0.82
22	Uridine	58-96-8	3.82	244.0695	[M-H]-, [M+FA-H]-,[M+Cl]-	C9H12N2O6	MS	0.1199	0.83	0.0117	0.70	0.0042	0.65
23	Phosphatidyl glycerol	61468-73-3	8.26	246.0505	[M+H]+, [M+Na]+	C6H15O8P	MS	0.9230	1.02	0.6039	1.09	0.0026	1.57
24	Glycerophosphocholine	28319-77-9	8.89	257.1028	[M+H]+,[M+Na]+, [2M+H]+,[2M+Na]+, [M+FA-H]-, Fragment	C8H20NO6P	MS/MS	0.0779	0.74	0.0190	0.65	0.0056	1.43
25	D-Glucose 6-phosphate	56-73-5	10.10	260.0297	[M-H]-	C6H13O9P	MS	0.0457	1.32	0.0463	1.32	0.0054	0.53
26	Inosine	58-63-9	5.16	268.0808	[M+H]+, [M+K]+, [2M+H]+, [2M+K]+, [M-H]-, [M+Cl]-,[2M+K-2H]-,Fragment	C10H12N4O5	MS2/MS	0.0051	0.76	0.0004	0.68	0.2103	0.90
27	Oleamide	301-02-0	4.30	281.2719	[M+H]+	C18H35NO	MS/MS	0.0062	0.50	0.0043	0.48	0.0370	0.64
28	Guanosine	118-00-3	6.38	283.0917	[M+H]+, [M-H]-	C10H13N5O5	MS	0.0607	0.69	0.0056	0.52	0.1032	1.27
29	Stearamide	124-26-5	1.30	283.2875	[M+H]+	C18H37NO	MS	0.0056	0.44	0.0020	0.36	0.0094	0.48
30	Glutathione	70-18-8	7.99	307.0838	[M+Na]+,[M+Na]+	C10H17N3O6S	MS	0.0418	0.69	0.0087	0.59	0.0125	0.61
31	N-	50854-94-	1.15	312.175	[M-H]-	C17H28O3S	MS	0.011	0.3	0.004	0.2	0.019	0.4

	Undecylbenzenesulfonic acid	9		9				7	4	8	5	5	0
32	α-Linolenoyl Ethanolamide	57086-93-8	1.39	321.2668	[M+Na]+	C20H35NO2	MS	0.6809	0.91	0.3889	1.18	0.0036	0.33
33	2-Dodecylbenzenesulfonic acid	——	1.14	326.1916	[M-H]-	C18H30O3S	MS	0.0097	0.38	0.0035	0.29	0.0153	0.43
34	docosapentaenoic acid	24880-45-3	0.68	330.2559	[M-H]-	C22H34O2	MS	0.0571	0.78	0.0081	0.68	0.0347	0.75
35	3-Deoxyvitamin D3	——	0.68	368.3443	[M+H]+	C27H44	MS	0.0064	0.58	0.0039	0.55	0.0302	0.68
36	Palmitoylcarnitine	2364-67-2	1.77	399.3349	[M+H]+	C23H45NO4	MS/MS	0.4846	1.16	0.6642	1.10	0.0097	1.66
37	Stearoylcarnitine	——	1.71	427.3662	[M+H]+	C25H49NO4	MS/MS	0.5075	1.20	0.7935	1.08	0.0090	1.86
38	1-Hydroxyvitamin D5	——	0.65	428.3654	[M+H]+	C29H48O2	MS	0.0267	0.64	0.0350	0.66	0.0103	0.58
39	Phe-Tyr-OH	——	8.68	436.1271	[M+H]+, [M+Na]+	C23H20N2O7	MS	0.7430	0.94	0.3257	0.83	0.0422	1.37
40	Lys-Trp-OH	——	8.97	440.1696	[M+H]+	C22H24N4O6	MS	0.0952	0.69	0.0436	0.62	0.0315	1.40
41	LysoPE(0:0/16:0)	——	5.12	453.2855	[M+H]+, [M-H]-	C21H44NO7P	MS/MS	0.5523	1.06	0.5894	1.06	0.0172	0.73
42	Cytidine diphosphate choline (CDPcholine)	987-78-0	11.16	488.1073	[M+H]+,[M+FA-H]-	C14H26N4O11P2	MS	0.3815	0.86	0.1898	0.79	0.0431	1.33
43	LysoPE(22:6/0:0)	——	5.02	525.2855	[M+H]+	C27H44NO7P	MS	0.2112	1.16	0.7954	1.03	0.0166	0.67
44	L-a-Lysophosphatidylserine	——	5.72	525.3067	[M-H]-	C24H48NO9P	MS	0.0253	1.52	0.0639	1.42	0.8261	0.95
45	N-Palmitoylsphingosine	24696-26-2	0.71	537.5121	[M+H]+, [M+Cl]-, Fragment	C34H67NO3	MS/MS	0.0103	0.66	0.0064	0.63	0.0389	0.73
46	DG(P-14:0/18:1)	——	5.37	550.4961	[M+H]+	C35H66O4	MS	0.0236	0.46	0.0122	0.39	0.0204	0.44
47	38:5	——	0.72	554.5063	[M+FA-H]-	C38H66O2	MS	0.1052	0.81	0.0122	0.69	0.0192	0.71

48	Oxidized glutathione	27025-41-8	11.18	612.1520	[M+H]+, [M+2H]2+,[M-H]-	C20H32N6O12S2	MS/MS	0.3681	1.20	0.0202	1.56	0.3235	0.78
49	PE(P-18:0/18:4)	——	3.46	723.5203	[M+H]+	C41H74NO7P	MS	0.0167	0.64	0.0064	0.58	0.0441	0.71
50	PE(13:0/22:1)	——	0.78	731.5465	[M+H]+	C40H78NO8P	MS	0.0056	0.47	0.0092	0.51	0.0134	0.54
51	PC(O-16:0/18:2)	——	3.78	743.5829	[M+H]+	C42H82NO7P	MS	0.0716	0.68	0.0121	0.54	0.0206	0.58
52	PC(P-18:1/16:0)	——	0.79	743.5829	[M+H]+	C42H82NO7P	MS	0.0045	0.39	0.0117	0.47	0.0165	0.50
53	PE(22:6/P-16:0)	——	0.79	747.5203	[M+H]+	C43H74NO7P	MS	0.0139	0.39	0.0303	0.47	0.0216	0.44
54	PE(P-18:1/20:4)	——	3.55	749.5359	[M+H]+	C43H76NO7P	MS	0.0175	0.57	0.0061	0.49	0.0175	0.57
55	PC(18:1/16:0)	——	3.91	759.5778	[M+H]+	C42H82NO8P	MS	0.0350	0.75	0.0082	0.68	0.0147	0.71
56	PC(22:1/12:0)	——	0.78	759.5778	[M+Na]+	C42H82NO8P	MS	0.0207	0.49	0.0148	0.46	0.0252	0.51
57	PS(16:1/18:0)	——	4.67	761.5207	[M+H]+	C40H76NO10P	MS	0.0265	0.72	0.0083	0.66	0.0456	0.75
58	PS(18:1/16:0)	——	5.02	761.5207	[M+H]+	C40H76NO10P	MS	0.0148	0.68	0.0055	0.63	0.0341	0.73
59	PC(16:0/20:4)	——	3.77	781.5622	[M+H]+	C44H80NO8P	MS	0.0239	0.54	0.0109	0.48	0.0313	0.57
60	PC(20:3/P-18:1)	——	0.77	793.5985	[M+H]+	C46H84NO7P	MS	0.0250	0.48	0.0239	0.48	0.0147	0.43
61	PC(16:0/22:6)	——	3.76	805.5622	[M+H]+	C46H80NO8P	MS	0.0612	0.65	0.0052	0.45	0.0646	0.66
62	PC(20:4/18:2)	——	0.78	805.5622	[M+H]+	C46H80NO8P	MS	0.0219	0.50	0.0176	0.48	0.0205	0.49
63	PI(16:0/16:0)	——	5.34	810.5258	[M-H]-	C41H79O13P	MS	0.0228	0.45	0.0089	0.35	0.0230	0.45

Notes: Statistical differences ($p < 0.05$ between control and BED47-treated testes samples were determined by T-test.

Table 3. Quantitative real-time PCR (qRT-PCR) primer sequences for selected genes.

Gene name	Primer Sequence (5'-3')		Accession number	Product length (bp)
	Forward	Reverse		
Myc	CCCTATTTCATCTGCGACGAG	GAGAAGGACGTAGCGACCG	NM_010849.4	185
Clu	AGCAGGAGGTCTCTGACAATG	GGCTTCCTCTAAACTGTTGAGC	NM_013492.3	164
Beta-actin	GGCTGTATTCCCCTCCATCG	CCAGTTGGTAACAATGCCATGT	NM_007393.5	154

Table 4. Top networks identified using Ingenuity Pathways Analysis software for significantly differential metabolites and proteins in testicular tissue.

Identification	Molecules in network	Score	Focus molecules	Top diseases and functions
1	26s Proteasome, actin, ATPase, calcineurin protein(s), calmodulin, CANX, caspase, CGN, CIT, Ck2, cytochrome C, DCTN1, Dst, ERK, estrogen receptor, FLOT1, Hsp70, Hsp90, hypoxanthine, LMNA, MYH10, MYH9, MYO18A, myosin, NASP,P-TEFb, proinsulin, RANGAP1, SPTAN1, SPTBN1, STXBP1, TTC4, VCP, VCPIP1, VIM	41	20	Cellular assembly and organization, cellular function and maintenance, connective tissue disorders
2	AChR, ALB, APOA1, CDP-choline, choline, cytochrome-c oxidase, Ggt, glutathione, glutathione disulfide, glutathione peroxidase, GOT, GSTM1, guanosine, HDL, hemoglobin, L-cysteine, Ldh (complex), LONP1, mitochondrial complex 1, NADPH oxidase, NFkB (complex), PI3K (family), PREP, pro-inflammatory cytokine, SDHB, serine protease, SERPINA3, Sod, succinate dehydrogenase, taurine, TF, trypsin, uracil, UTP3, xanthine	36	18	Free radical scavenging, amino acid metabolism, molecular transport
3	Acetyl-L-carnitine, alpha catenin, calpain, COL12A1, COL6A1, COL6A2, Col6a4, COL6A5, collagen, collagen type I, collagen type IV, collagen type VI, collagen(s), creatine kinase, CTSV, cyclin A, D-erythro-C16-ceramide, DCN, ERK1/2, F13A1, fibrin, fibrinogen, growth hormone, HSPG2, LAMA1, laminin (complex), Mmp, MYH11, PDGF BB, phosphocreatine, Raf, RBP1, Rock, Tgf beta, WNK4	31	16	Connective tissue, developmental, hereditary disorder
4	Acetylcholine, AKAP12, Akt, AMPK, ANXA6, Ap1, C1q, CALR, CaMKII, CCAR2, CLGN, CLU, Dync1i2, F actin, IgG, IgG1, IL1, IL12 (complex), immunoglobulin, inosine, LCP1, LDHB, LDL, LIPE, Mek, MVP, Ngf, p70 S6k, PI3K (complex), Pld, PRKAA, SAMHD1, spectrin, STAT5a/b, TGM2	28	15	Cardiovascular system development and function, organ development, cellular assembly and organization
5	ABCF3, ACOT1, Akr1b7, ATP10A, BCL6, CABS1, CFAP20, CFAP36, CSMD1, FILIP1, FRG1, HNF4A, HNRNPL, HOOK2, HSD17B2, HSD17B8, KPNA2, miR-1291 (and other miRNAs w/seed GGCCCUG), mir-28, miR-5624-3p (and other miRNAs w/seed UAAGGCA), NTRK1, Ntrk1 dimer, PGM2, PGP, RAC1, SART3, SHTN1, SUPV3L1, TCF4, TENM4, THAP2, TMC6, TTC12, VWA5A, WDR13	21	12	Cell morphology, cellular assembly and organization, cellular function and maintenance

6	ANXA2, CD3, CHD4, Creb, cytokine, focal adhesion kinase, FSH, GIT1, glucose-6-phosphate, Gsk3, Hbb-b1, histone h3, histone h4, IDH1, insulin, Jnk, MAP4, Mapk, NMDA receptor, P38 MAPK, p85 (pik3r), Pka, Pkc(s), PKM, PLC, Rac, RAS, Ras homolog, RNA polymerase II, SAFB, Sos, SRC (family), TCR, uridine, Vegf	17	10	Cancer, neurological disease, organismal injury and abnormalities
7	(all Z)-7,10,13,16,19-docosapentaenoic acid, 2210010C04Rik, 5-hydroxydecanoic acid, ACSF2, adenosine triphosphate, AHCY, ARMC3, basic calcium phosphate crystal, Bcl9-Cbp/p300-Ctnnb1-Lef/Tcf, Ca2, CAD, CDK2, CDK2-CyclinE, Convulxin, D-aspartic acid, Dstn/Dstnl1, FOS, GJA1, Gm-Csf receptor, Hnrnph1, Hrnr, K, Ldha/RGD1562690, LOC100359583/Ptma, miR-6758-5p (and other miRNAs w/seed AGAGAGG), MYC, nicotinate adenine dinucleotide phosphate, NPS, NUDC, oleylamide, Ppp1r15a, RMDN1, sn-glycero-3-phosphocholine, TSNAX, Uba52	15	9	Amino acid metabolism, molecular transport, small molecule biochemistry
